# Pathogenic bacterial profile and antimicrobial susceptibility patterns in odontogenic facial cellulitis among Egyptian patients: A cross-sectional study

**DOI:** 10.1038/s41598-026-64016-7

**Published:** 2026-08-06

**Authors:** Fatma A.N. Abouel Maaty, Sara El Moshy, Azza Gramoun, Mohamed Abdelmoteleb, Aala’a Emara, Maha H. Bashir, Marwa M. S. Abbass

**Affiliations:** 1 Oral Biology Department, Faculty of Dentistry, Cairo University, Cairo, Egypt; 2 Consultant at Azante Medical Reporting and Analytics, Munich, Germany; 3 Botany Department, Faculty of Science, Mansoura University, Mansoura, Egypt; 4 Oral and Maxillofacial Surgery Department, Faculty of Dentistry, Cairo University, Cairo, Egypt; 5 Oral Biology Division, Basic Sciences Department, Faculty of Dentistry, GUC University, Cairo, Egypt

**Keywords:** Odontogenic facial cellulitis, Antibiotic resistance, 16S rRNA sequencing, Egypt, Polymicrobial infection, Diseases, Medical research, Microbiology

## Abstract

Odontogenic facial cellulitis is the most common facial space infection worldwide, originating from untreated dental caries and progressing to life-threatening complications. This stems from the polymicrobial nature of the disease, along with the widespread and over-the-counter misuse of antibiotics in Egypt. This study aims to identify the pathogenic bacterial species involved in odontogenic facial cellulitis in an Egyptian patient sample. Forty-four samples were collected from patients presenting with odontogenic facial cellulitis. Bacterial profiles were investigated using selective media, Gram staining/colony identification, biochemical tests, and Clinical and Laboratory Standards Institute standardized antibiotic susceptibility testing. Resistant strains (≥ 3 agents) were identified using 16 S rRNA sequencing and deposited in the NCBI GenBank. Odontogenic cellulitis originated primarily from lower posterior teeth, specifically the lower first molar, and spread commonly in the submandibular and submental spaces. Regarding bacterial profile, the Gram-positive cocci were the predominant ones. Complete resistance to metronidazole was reported, while previous antibiotic administration showed no statistically significant association with susceptibility to amoxicillin and clindamycin. Among multidrug-resistant isolates, 16 S rRNA sequencing identified predominantly *Bacillus*, *Enterococcus*, and *Staphylococcus* species. Two isolates showed < 90% identity to known species, suggesting possible novel taxa and informing empirical therapy through phenotypic resistance profiles.

**Supplementary Information:**

The online version contains supplementary material available at 10.1038/s41598-026-64016-7.

## Introduction

Odontogenic facial cellulitis is potentially a life-threatening soft tissue infection stemming from dental pathology. Commonly, the infection starts as an untreated dental cavity that leads to pulpitis with subsequent periapical infection and abscess formation. Pathogenesis involves bacterial invasion from the dental alveolus to the adjacent subcutaneous tissue. This process allows the infection to spread through facial tissue planes, leading to widespread cellulitis^1^. Clinically, this pathology shows up as a fast-growing, diffuse swelling of the cheek or jaw. It is often associated with tenderness together with systemic symptoms such as fever and malaise^2^.

Epidemiological reports revealed that approximately 5% to 30% of the severe cervicofacial cellulitis cases in hospitals were of odontogenic origin^3,4^. The aggressive and rapid progression of this disease renders it a critical public health concern that urges prompt diagnosis and management. Severe complications of odontogenic facial cellulitis include necrotizing mediastinitis. This happens when the infection spreads along the cervico-facial planes into the thoracic cavity, which can lead to high rates of illness and fatality. Additionally, the infection can spread to the orbit, causing orbital cellulitis that may result in permanent vision loss^5^. Another rare but devastating complication is the intracranial empyema, pointing out the critical need for early intervention^6^.

The most common microorganisms involved are *Streptococcus*, which are Gram-positive aerobic organisms, and strict Gram-negative organisms such as *Prevotella*, *Porphyromonas*, and *Fusobacterium*. One study reported that the most frequently bacteria in odontogenic infections are *Streptococcus mutans* (24.5%), *Porphyromonas gingivalis* (23.6%), *Porphyromonas endodontalis* (18.2%), *Streptococcus salivarius* (10.1%), and *Streptococcus sanguis* (8.2%)^2^. Another study study has identified *Streptococci* and *Staphylococcus aureus* (*S. aureus*) as the leading pathogens in cellulitis patients^7^. Egyptian reports have highlighted that about 36% of odontogenic infections are due to pure aerobic bacteria, 5% to pure anaerobic bacteria, and 53% to mixed aerobic-anaerobic flora. Such microbial diversity highlights the complexity of the microbial environment and the need for antibiotics that work against both types^8-10^. *Klebsiella pneumoniae* (*K. pneumoniae)* is a member of the ESKAPE group of pathogens, which are recognized for their ability to evade antimicrobial therapy and contribute substantially to the global burden of antimicrobial resistance. *K. pneumoniae* is frequently associated with multidrug resistance, including the potential to produce extended-spectrum β-lactamases (ESBLs), posing significant therapeutic challenges. These characteristics highlight the importance of antimicrobial resistance surveillance and antibiotic stewardship in the management of odontogenic infections^11^.

The polymicrobial nature of odontogenic facial cellulitis reflects the complexity of the oral flora that can contribute synergistically to infection severity and progression. Among these bacteria, *Staphylococcus aureus* stands out as a frequent pathogen known for its role in orofacial infections and its increasing resistance to antibiotics^9^. *Proteus mirabilis*, although less commonly emphasized, is also relevant due to its virulence factors and capacity to cause complicated soft tissue infections, including those of odontogenic origin^12^. Their detection in polymicrobial infections provides deeper insight into the bacterial spectrum involved, especially in Egyptian patients, where such data remain essential to optimize antibiotic therapy and clinical outcomes.

Both surgical interventions and medicinal approaches are indispensable for the management of odontogenic facial cellulitis. The surgical part typically constitutes incision and drainage, along with broad-spectrum antibiotics^13^. Antimicrobial resistance (AMR) continues to pose a growing global public health challenge. Current projections estimate that AMR-associated deaths could exceed 39 million cumulatively between 2025 and 2050, with the annual number of deaths directly attributable to drug-resistant infections expected to increase from approximately 1.14 million in 2021 to nearly 1.91 million by 2050 ^14^. In Egypt, trading antibiotics without official authorization constitutes a pivotal problem leading to massive antibiotic misuse and overuse. These misconducts strongly contribute to the emergence and spread of new resistant microbial strains^14^.

Resistance to commonly used antibiotics such as penicillin and amoxicillin has been increasingly documented in Egypt^15^. Even though combinational therapy involving amoxicillin and clavulanic acid (a beta-lactamase inhibitor) remains effective against many resistant strains, resistance to clindamycin is on the rise. This mandates wise and careful use of this antibiotic^16,17^.

Despite the clinical significance of odontogenic facial cellulitis, there is a lack of detailed data about the specific bacterial species involved and their antibiotic resistance profiles among the Egyptian population. Furthermore, antibiotic sensitivity patterns in Egypt showed significant differences as compared to regional and global published data^18^. Such a gap limits the clinicians’ ability to select the most effective antimicrobial therapy and to develop proper strategies to combat resistance. Accordingly, this study aims to address this gap by identifying the harmful and resistant bacteria responsible for odontogenic facial cellulitis in a convenient sample of the Egyptian patients^18^. To our knowledge, this is the first Egyptian study to integrate conventional microbiological identification, antimicrobial susceptibility testing, and 16 S rRNA sequencing of multidrug-resistant isolates, providing a comprehensive characterization of the bacterial profile of odontogenic facial cellulitis and locally generated evidence to support empirical antibiotic therapy.

## Materials and methods

### Ethical considerations

This study was conducted in accordance with the Declaration of Helsinki, and according to the guidelines of the Research Ethics Committee, Faculty of Dentistry, Cairo University (protocol approval no. 25322). Valid informed consents were obtained from all participants after explaining the aims of the study.

### Sample size

The sample size for the current study was statistically calculated to determine the minimum number of cases required to accurately identify and characterize the different bacterial species associated with odontogenic facial cellulitis among adult Egyptian patients. A broad-based review of previously published literature was conducted, disclosing that the reported prevalence of bacterial isolates for Proteus mirabilis was 2.27% while that for staphylococcus aureus was 34.1% ^19^. Accordingly, it was estimated that a total of 44 clinical samples would be convenient to detect a 1.96% variation in bacterial distribution with a statistical power of 80%, while maintaining the level of significance (alpha error) at 0.05. The sample size calculation was performed using the G*power program^20^.

### Sample collection

The study was conducted from April 2022 to April 2023. Samples were collected from 44 patients presenting with odontogenic facial cellulitis in the emergency room of the Oral and Maxillofacial Department, Faculty of Dentistry, Cairo University. Patients were registered in the study based on defined inclusion and exclusion criteria. The inclusion criteria for this study include patients from both genders who were diagnosed with odontogenic facial cellulitis and aged between 18 and 60 years. Enrollment in the study was permitted regardless of any prior administration of oral antibiotics. Whereas the exclusion criteria for this study were patients with facial cellulitis of non-odontogenic origin, such as infections arising from surgical wounds, upper respiratory tract infections, infected cysts, or neoplasms. Additionally, individuals who refused to sign the consent form, pregnant patients, those younger than 17 years or older than 60 years, patients who had received intramuscular or intravenous antibiotics, immunocompromised individuals, and patients who had already undergone incision and drainage were all excluded from participation.

Following a thorough clinical examination, extraoral sites were prepared with povidone iodine before sample collection. The sampling of pus or exudate was performed by a senior oral and maxillofacial surgeon during incision and drainage procedure, strictly following aseptic conditions using a sterile swab. Sampling during incision and drainage allows direct access to the purulent exudate, and this provides the highest yield of the causative bacteria for accurate identification and sensitivity testing. Throughout specimen collection, handling, microbiological culture, DNA extraction, and molecular analyses, sterile instruments and consumables were used. All procedures were performed under aseptic laboratory conditions to minimize the risk of environmental contamination. Once swelling and acute symptoms subsided, the infected tooth was commonly treated by extraction or root canal treatment. The swabs were sent immediately at 4 °C in a cool box with precooled gel packs to the microbiology laboratory for culture and sensitivity.

### Microbiological analysis

#### Isolation, staining, and bacterial identification

Clinical specimens were inoculated onto blood agar, Vogel–Johnson agar, Mueller–Hinton agar, and tryptic soy agar immediately upon receipt in the microbiology laboratory. The inoculated media were incubated aerobically at 37 °C for 48–72 h, and culture plates were examined daily for bacterial growth for up to 5 days. Isolates obtained from positive cultures were identified in the present study based on their colony morphology and Gram-staining characteristics according to the study protocol^21^. Following primary isolation, bacterial culture samples were divided into three aliquots: one for detecting the presence of S*taphylococcus aureus* and *Proteus mirabilis*, one for antibiotic sensitivity testing, and the last one for 16 S rRNA sequencing.

### Detection of staphylococcus aureus versus proteus mirabilis

Bacterial isolates recovered from the culture plates were subjected to confirmatory biochemical testing according to the study protocol. Identification of *Staphylococcus aureus* was confirmed using the catalase test and blood hemolysis on blood agar, whereas *Proteus mirabilis* was identified using urease and indole tests. For quantitative analysis, the bacterial load was expressed as colony-forming units per milliliter (CFU/mL) and were calculated by dividing the colony count by the plated volume and multiplying by the inverse dilution factor^22,23^.

### Antimicrobial susceptibility test

**Antimicrobial susceptibility testing** was performed in the present study using the agar well diffusion method. Standardized bacterial suspensions prepared from the recovered isolates were inoculated onto Mueller–Hinton agar plates. Wells were then created in the agar, filled with the selected antibiotic solutions, and the plates were incubated under the study conditions. Following incubation, the diameters of the inhibition zones surrounding each well were measured in millimeters and interpreted according to the study protocol^24^. The antimicrobial panel comprised amoxicillin, clindamycin (Dalacin C), metronidazole (Flagyl), and novobiocin. Amoxicillin, clindamycin, and metronidazole were selected because they are commonly used in the management of odontogenic infections. Novobiocin was included as part of the standardized antimicrobial susceptibility panel used during the study period. Interpretation of inhibition zones was performed according to the Clinical and Laboratory Standards Institute (CLSI) guidelines.

### DNA extraction, PCR amplification, and 16 S rRNA gene sequencing

Resistance to three or more of the tested antibiotics was recorded in 14 samples. Consequently, they were selected for 16 S rRNA sequencing to determine their taxonomic identity. Genomic DNA was extracted from pure bacterial cultures using the QIAamp^®^ DNA Mini kit (QIAGEN, Hilden, Germany). DNA was purified through silica-membrane spin columns according to the manufacturer’s instructions, and DNA quality and concentration were subsequently assessed by agarose gel electrophoresis and spectrophotometric quantification. 16 S rRNA gene was amplified utilizing the universal primers 27 F (5’-AGAGTTTGATCCTGGCTCAG-3’) and 1492R (5’-GGYTACCTTGTTACGACTT-3’), addressing the V1–V9 regions. PCR amplicons were purified using a Montage PCR Clean-up Kit (Millipore) to remove residual primers and dNTPs prior to sequencing. Purified amplicons were sequenced using the BigDye Terminator v3.1 Cycle Sequencing Kit (Applied Biosystems) on an automated DNA sequencer. Subsequently, as an assessment of the evolutionary relationships between the isolates, phylogenetic trees were constructed for each one of them. As a final step, the obtained sequences were submitted to the GenBank database for identifying strains and an accession number for each isolate was acquired.

### Phylogenetic analysis

Phylogenetic trees were constructed for each isolate using the maximum-likelihood (ML) method implemented in MEGA-11 software^25^. MEGA-11 software was used, and sequence alignments were independently performed using both ClustalW and MUSCLE to assess alignment robustness. First the sequences were aligned using ClustalW^26^. The best-fitting nucleotide substitution model was selected in MEGA-11 using the Bayesian Information Criterion (BIC). The K2 + G + I model (Kimura two-parameter with gamma distribution and invariant sites) exhibited the lowest BIC score and was subsequently used to reconstruct maximum-likelihood phylogenetic trees. Branch support was assessed using 100 bootstrap replicates. Second, the sequences were subsequently realigned using MUSCLE, and the same analytical procedure was applied to reconstruct maximum-likelihood phylogenetic trees and assess branch support^27^.

### Data analysis

Stratification of patients by age was done into two groups: Group I (18–30 years) and Group II (31–60 years). The exact cut-offs were chosen to reflect the clinically relevant adult stages, ensuring adequate sample sizes, aligning with prior research, and accounting for age-related differences in immune function, skin microbiota, and comorbidities affecting infection patterns^28^.

Antibiotic susceptibility results were classified according to standard interpretive categories as “sensitive,” “intermediate,” and “resistant,” based on zone diameter breakpoints. Isolates classified as “intermediate” were combined with the “resistant” category for analytical purposes. This approach is justified because the intermediate category, which acts as a buffer zone, contains isolates with decreased susceptibility that may not consistently respond to standard dose schedules. In clinical practice and research, combining “intermediate” and “resistant” is a conservative strategy that avoids overestimating an antibiotic’s expected efficacy, leading to a more thorough assessment of the burden of antimicrobial resistance^29^.

Patients were classified as “resistant” if they had two isolates, one of which was resistant to the tested antibiotics and the other was sensitive. This approach was used to ensure a conservative estimate of the prevalence of antibiotic resistance, avoid underestimating resistant rates, and comply with clinical decision-making where the most resistant pathogen is often used to determine therapy^30^.

Infections involving the buccal and canine spaces were combined into a single group, as these spaces are anatomically contiguous and frequently involved together in facial cellulitis. On a similar basis, infections involving the submandibular and submental spaces were grouped due to their close anatomical proximity and close clinical management. This grouping was made to enhance statistical power and reflect clinical practice, where these spaces are commonly affected concurrently and share similar treatment protocols^31,32^.

### Statistical analysis

Data were statistically described as frequencies (number of cases) and percentages. Because all outcomes were categorical, Chi-square tests were used to evaluate associations between variables. P values of less than 0.05 were considered statistically significant. All statistical calculations were conducted using the computer program IBM SPSS (Statistical Package for the Social Sciences; IBM Corp, Armonk, NY, USA) release 27 for Microsoft Windows.

## Results

### Demographics

Among the 44 participants enrolled, 28 were males (63.64%), and 16 were females (36.36%). Of these, 24 (54.55%) were in age group GI and 20 (45.45%) in age group GII Table (1).

### Involved teeth and their relationship with gender and age

The majority of participants in this study 31 (70.45%) had infections related to lower posterior teeth, specifically the lower first molar 26 (59.09%), lower first and second molars 2 (4.55%), lower third molar 2 (4.55%), and lower second premolar 1 (2.27%). Only 1 (2.27%) participant had an infection related to the lower lateral incisor. Infections involving upper anterior teeth were observed in 7 (15.91%) participants, mainly affecting the upper canines 5 (11.36%), with isolated cases in the upper central and lateral incisors 1 (2.27%) and upper lateral incisors 1 (2.27%). Upper posterior teeth were involved in 5 (11.36%), including the upper first molar 4 (9.09%) and upper first premolar 1 (2.27%). Among males and females, the majority had infections in the lower posterior teeth 19 (67.86%) males; 12 (75%) females. Similarly, most patients in age groups GI 15 (62.50%) and GII 16 (80%) were affected in the lower posterior region Table 1.

**Table 1:** Patient demographics and number of involved teeth.

Parameter	Categories *n* (%)			
Age (years)	GI (19–30 yrs)		GII (31–60 yrs)	
	24 (54.55%)		20 (45.45%)	
Gender	Males		Females	
	28 (63.64%)		16 (36.36%)	
Tooth involved n (%)				
Lower anterior teeth 1(2.27%)	Lower lateral incisor 1(2.27%)			
Lower posterior teeth 31(70.45%)	Lower first and second molars 2(4.55%)			
	Lower first molar 26(59.09%)			
	Lower second premolar 1(2.27%)			
	Lower third molar 2(4.55%)			
Upper anterior teeth 7(15.91%)	Upper Canine 5(11.36%)			
	Upper central, lateral incisors 1(2.27%)			
	Upper lateral incisor 1(2.27%)			
Upper posterior teeth 5(11.36%)	Upper first premolar 1(2.27%)			
	Upper first molar 4(9.09%)			
Tooth involved n (%)	Gender		Age	
	Males	Females	GI	GII
Lower anterior teeth 1(2.27%)	1(3.57%)	0	1(4.17%)	0
Lower posterior teeth 31(70.45%)	19(67.86%)	12(75%)	15(62.50%)	16(80%)
Upper anterior teeth 7(15.91%)	3(10.71%)	4(25%)	3(12.50%)	4(20%)
Upper posterior teeth 5(11.36%)	5(17.86%)	0	5(20.83%)	0

### Microbiological evaluation

*Staphylococcus aureus* was detectable in 8 (18.18%) patients, while 36 (81.82%) was negative. *Proteus mirabilis* was detected in 4 (9.09%), while 40 (90.91%) were negative (Table 2). A single isolate was found in the majority of male patients 22 (78.57%), and half of female patients 8 (50%). By age, most patients in groups GI 18 (75%) and GII 12 (60%) had a single isolate. There were no statistically significant differences between gender or age groups Table (3). Among patients with one or two isolates (*n* = 34), most showed cocci morphology, 25 (73.5%) bacilli were identified in 5(14.74%), while a smaller portion, 4 (11.76%) isolates exhibited a combination of both cocci and bacilli morphologies. In terms of Gram stain characteristics, 31 (91.18%) isolates were Gram-positive. Conversely, 3 (8.82%) isolates were Gram-negative Table 2.

**Table 2:** S*taphylococcus aureus* and *Proteus mirabilis* detection, Bacterial isolate, bacterial morphology, and Gram stain.

Parameter	Categories *n* (%)					
	Detected			Negative		
S*taphylococcus aureus*	8(18.18%)			36(81.82%)		
*Proteus mirabilis*	4(9.09%)			40(90.91%)		
Bacterial isolate n (%)	Gender		P value	Age		P value
	Males	Females		GI	GII	
2 isolates 4 (9.09%)	1 (3.57%)	3 (18.75%)	0.100	1(4.17%)	3(15%)	0.396
Single isolate 30 (68.18%)	22 (78.57%)	8 (50%)		18 (75%)	12(60%)	
Negative 10 (22.72%)	5 (17.86%)	5 (31.25%)		5 (20.84%)	5(25%)	
Bacterial morphology n (%)						
Cocci	25 (73.5%)					
Bacilli	5 (14.74%)					
Cocci and bacilli	4 (11.76%)					
Gram Stain n (%)						
Gram Positive	31 (91.18%)					
Gram Negative	3 (8.82%)					

### Region of facial cellulitis

**Fig. 1:**
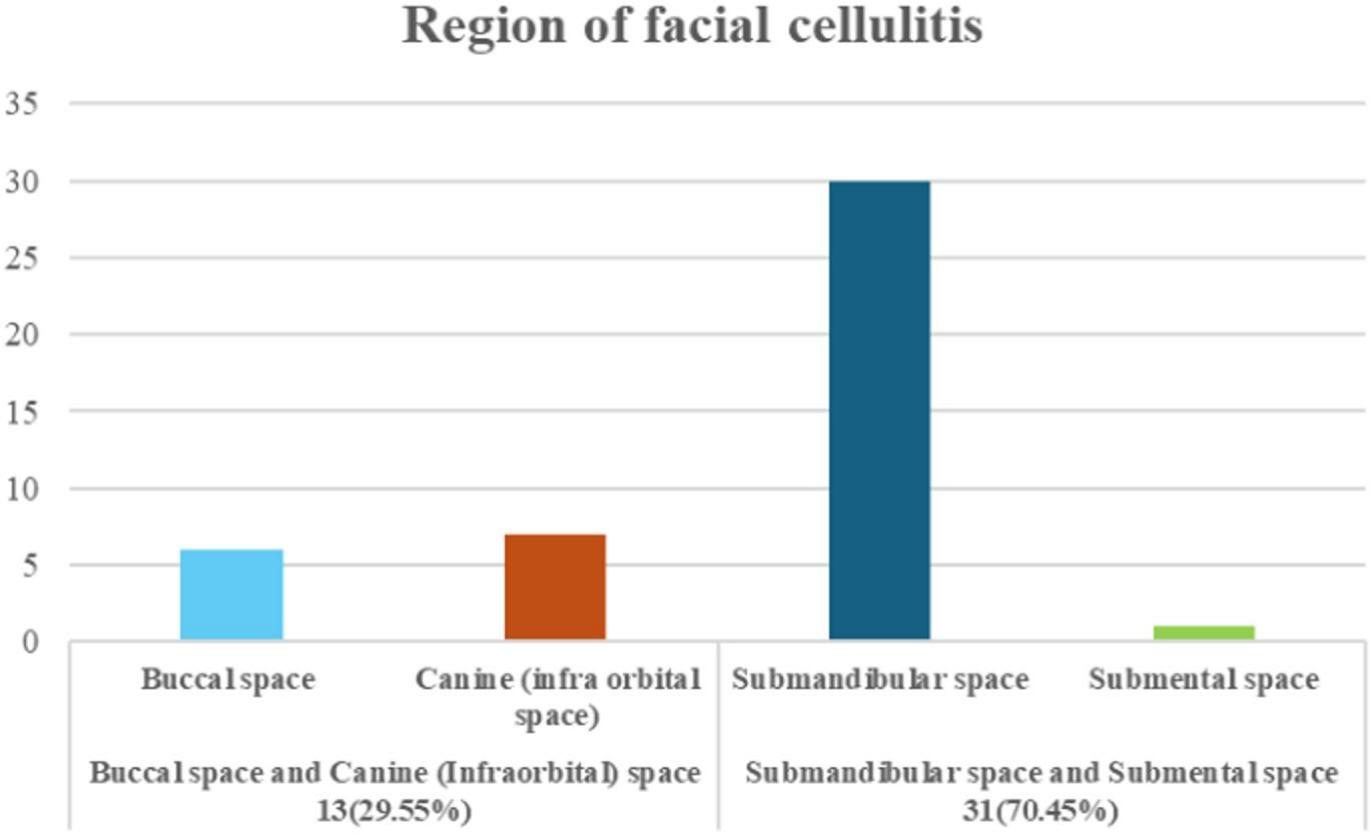
Bar chart showing region of facial cellulitis.

Most patients had cellulitis in the Submandibular and Submental spaces. The total number of patients with Submandibular and Submental spaces involvement was 31 (70.45%), distributed as follows 30 (68.18%) in the Submandibular and only 1 (2.27%) in the Submental space. Cellulitis in the Buccal and Canine (Infraorbital) spaces was less common, affecting 13 (29.55%) patients, 7 (15.91%) in the Canine, and 6 (13.64%) in the Buccal space. The majority of both males 20 (71.43%), and females 11 (68.75%) had cellulitis in the Submandibular and Submental spaces, with no significant gender difference. Similarly, most patients in age groups GI 16 (66.67%) and GII 15 (75%) were affected in these spaces, with no significant difference between age groups Table 3; Fig. 1. Among patients with cellulitis in the Buccal and Canine spaces, 8 (26.67%) had a single isolate, while 22 (73.33%) of those with Submandibular and Submental cellulitis had a single isolate; the difference was not statistically significant Table 3.

**Table 3:** Region of facial cellulitis.

Parameter	Categories *n* (%)									
Buccal space and Canine (Infraorbital) space 13(29.55%)	Buccal space				6(13.64%)					
	Canine (Infraorbital) space				7(15.91%)					
Submandibular space and Submental space 31(70.45%)	Submandibular space				30(68.18%)					
	Submental space				1(2.27%)					
Region of facial cellulitis n (%)	Gender		P value	Age		P value	Bacterial Isolate			**P value**
	Males	Females		GI	GII		Two isolates	Single isolate	Negative	
Buccal space and Canine (Infraorbital) space 13(29.55%)	8(28.57%)	5(31.25%)	0.851	8(33.33%)	5(25%)	0.546	0	8(26.67%)	5(50%)	0.149
Submandibular space and Submental space 31(70.45%)	20(71.43%)	11(68.75%)		16(66.67%)	15(75%)		4(100%)	22(73.33%)	5(50%)	

### Previous antibiotic administration

Most participants 36 (81.82%) had previously used antibiotics for managing facial cellulitis. Antibiotic use was reported by 20 (71.43%) males and 16 (100%) females, showing a significant difference (*P* = 0.018). In age groups GI and GII, 18 (75%) patients had prior antibiotic treatment, with no significant difference between groups Table 4.

### Antibiotic susceptibility test

**Fig. 2:**
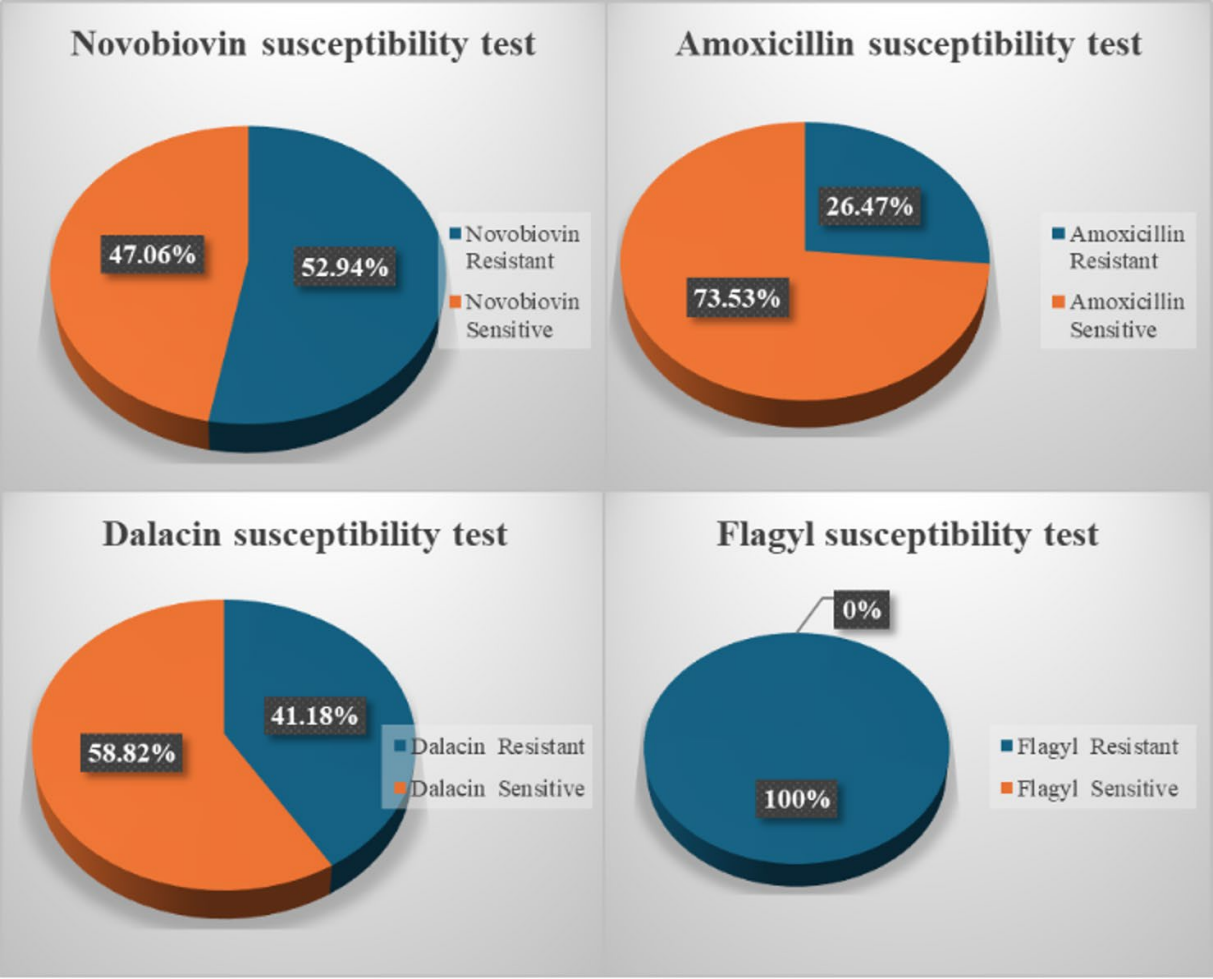
Pie chart showing the antibiotic susceptibility test.

Among the tested antibiotics, all patients (100%) showed resistance to Flagyl, with no cases of sensitivity detected. For Amoxicillin, the majority of patients (73.53%) were sensitive, while 26.47% were resistant. In the case of Dalacin, (58.82%) of patients were sensitive and (41.18%) were resistant. For Novobiocin, (52.94%) of patients exhibited resistance, while (47.06%) were sensitive, Fig. 2.

### Antibiotic susceptibility test vs. Patients’ demographics

Among patients with one or two bacterial isolates (*n* = 34), 100% (23 males and 11 females) showed resistance to Flagyl. The majority were sensitive to Amoxicillin (25) 73.53% andDalacin (20), 58.82%, while 18 (52.94%) exhibited intermediate resistance or resistance to Novobiocin. By gender, 100% of both males (23) and females (11) were resistant to Flagyl. For Novobiocin, 13 (56.52%) of males were sensitive, whereas 8 (72.73%) of females showed intermediate resistance or resistance. Regarding Amoxicillin, 19 (82.61%) of males and 6 (54.55%) of females were sensitive. Sensitivity to Dalacin was observed in 13 (56.52%) of males and 7 (63.64%) of females. No significant gender differences were found for Novobiocin, Amoxicillin, or Dalacin susceptibility. By age group, 100% of GI 19 and GII 15 showed resistance to Flagyl. Intermediate resistance or resistance to Novobiocin was observed in (10) 66.67% of GII and (8) 42.11% of GI, with no significant difference. Sensitivity to Amoxicillin was higher in GI (17) 89.47% than GII (8) 53.33%, showing a significant difference (*P* = 0.018). Similarly, Dalacin sensitivity was greater in GI (14) 73.68% than GII (6) 40.00% with a significant difference (*P* = 0.048) Table 4.

### Antibiotic susceptibility test vs. region of facial cellulitis and Previous antibiotic administration

Among patients with Submandibular and Submental cellulitis, (19) 73.08% were sensitive to Amoxicillin and (15) 57.69% to Dalacin, while (14) 53.85% showed intermediate resistance or resistance to Novobiocin. For those with Canine (Infraorbital) and Buccal cellulitis, (6) 75.00% were sensitive to Amoxicillin, (5) 62.50% to Dalacin, and an equal number showed sensitivity and resistance to Novobiocin. No significant differences were observed between cellulitis location and antibiotic susceptibility for Novobiocin, Amoxicillin, or Dalacin. Regarding previous antibiotic use, (17) 65.38% of participants reported intermediate resistance or resistance to Novobiocin, whereas (7) 87.50% of those without prior antibiotic use were sensitive to it, showing a significant difference (*P* = 0.009). Sensitivity to Amoxicillin (17) 65.38% and Dalacin (14) 53.85% was reported among those with prior antibiotic use, but differences were not statistically significant. All participants (26) 100% with previous antibiotic use, were resistant to Flagyl Table 4.

**Table 4:** Previous antibiotic administration, antibiotic susceptibility test vs. region of facial cellulitis, previous antibiotic administration, and patients’ demographics.

Parameter		Categories *n* (%)											
Previous antibiotic administration n (%)		Gender		P value	Age		P value						
		Males	Females		GI	GII							
Yes 36(81.82%)		20 (71.43%)	16 (100%)	0.018*	18 (75%)	18 (75%)	0.199						
No 8 (18.18%)		8 (28.57%)	0		6 (25%)	2 (10%)							
Antibiotic susceptibility test n (%)		Region of facial cellulitis			Previous antibiotic administration			Gender		P value	Age		P value
		Canine (Infraorbital) and Buccal spaces	Submandibular and Submental spaces		Yes	No		Males	Females		GI	GII	
Novobiocin	Intermediate resistance & resistance 18(52.94%)	4(50.00%)	14(53.85%)	0.849	17(65.38%)	1(12.50%)	0.009*	10(43.48%)	8(72.73%)	0.110	8(42.11%)	10(66.67%)	0.154
	Sensitive 16(47.06%)	4(50.00%)	12(46.15%)		9(34.62%)	7(87.50%)		13(56.52%)	3(27.27%)		11(57.89%)	5(33.33%)	
Amoxicillin	Intermediate resistance & resistance 9(26.47%)	2 (25.00%)	7 (26.92%)	0.914	9(34.62%)	0	0.052	4(17.39%)	5(45.45%)	0.083	2(10.53%)	7(46.67%)	0.018*
	Sensitive 25(73.53%)	6 (75.00%)	19 (73.08%)		17(65.38%)	8(100%)		19(82.61%)	6(54.55%)		17(89.47%)	8(53.33%)	
Dalacin	Intermediate resistance & resistance 14(41.18%)	3(37.50%)	11(42.31%)	0.809	12(46.15%)	2(25%)	0.288	10(43.48%)	4(36.36%)	0.693	5(26.32%)	9(60.00%)	0.048*
	Sensitive 20(58.82%)	5(62.50%)	15(57.69%)		14(53.85%)	6(75%)		13(56.52%)	7(63.64%)		14(73.68%)	6(40.00%)	
Flagyl	Intermediate resistance & resistance 34(100%)	8(100%)	26(100%)		26(100%)	8(100%)		23(100%)	11(100%)		19(100%)	15(100%)	
	**Sensitive**	0	0		0	0		0	0		0	0	

### Identification of bacterial isolates

Table 5 revealed a diverse collection of bacterial isolates, predominantly belonging to the genera *Bacillus*, *Staphylococcus*, *Enterococcus*, and *Klebsiella*. Most of the isolates demonstrated high percent identity values (ranging from 97.2% to 99.63%) when compared to their reference strains in GenBank, indicating a reliable species-level identification. *Bacillus* and *Enterococcus*species were the most frequently identified ones. This suggests their prevalence in the sampled environment. Two isolates (3 and 10) showed less than 90% identity and could not be confidently matched to known organisms. This may point out the presence of novel or less-characterized bacterial species. As for enhancing the traceability, the accession numbers for each match were registered in NCBI GenBank listed in Table 5, allowing further sequence verification. The high identity scores given support the accuracy of the identifications, while the unidentified isolates are considered as potential areas for further investigation and sequencing. The Phylogenetic trees in the supplementary material S1 were constructed using the highly matched 16 S rRNA gene sequences to the twelve strains. It illustrates taxonomy and evolutionary relationships among various bacterial species and their related taxa, suggesting a high degree of genetic similarity.

**Table 5:** Isolates’ identification and accession number.

Isolate No.	Best matched organism	Percent identity	GenBank	GenBank accession number
1	*Bacillus paramycoides* strain MCCC1A04098	98.88	https://www.ncbi.nlm.nih.gov/nuccore/OR365284	OR365284
2	*Bacillus cereus* strain JCM 2152	99.35	https://www.ncbi.nlm.nih.gov/nuccore/OR365495	OR365495
3	NA	< 90%		NA
4	*Bacillus fungorum* strain 17-SMS-01	99.63	https://www.ncbi.nlm.nih.gov/nuccore/OR365496	OR365496
5	*Staphylococcus aureus* strain NBRC 100,910	99.25	https://www.ncbi.nlm.nih.gov/nuccore/OR365499	OR365499
6	*Staphylococcus schweitzeri* strain DSM 28,300	99.4	https://www.ncbi.nlm.nih.gov/nuccore/OR365504	OR365504
7	*Staphylococcus simiae* strain CCM 7213 = CCUG 51,256	97.2	https://www.ncbi.nlm.nih.gov/nuccore/OR365510	OR365510
8	*Klebsiella pneumoniae* subsp. ozaenae strain ATCC 11,296	98.04	https://www.ncbi.nlm.nih.gov/nuccore/OR365511	OR365511
9	*Enterococcus faecium* strain NBRC 100,485	98.63	https://www.ncbi.nlm.nih.gov/nuccore/OR365514	OR365514
10	NA	< 90%		NA
11	*Bacillus licheniformis* strain DSM 13	98.44	https://www.ncbi.nlm.nih.gov/nuccore/OR365534	OR365534
12	*Enterococcus faecium* strain LMG 11,423	98.09	https://www.ncbi.nlm.nih.gov/nuccore/OR365662	OR365662
13	*Bacillus sanguinis* strain BML-BC004	98.93	https://www.ncbi.nlm.nih.gov/nuccore/OR365663	OR365663
14	*Enterococcus asini* strain NBRC 100,681	98.99	https://www.ncbi.nlm.nih.gov/nuccore/OR365665	OR365665

NA. Isolate has sequence identity < 90% to public strains.

## Discussion

Odontogenic facial cellulitis in the Egyptian population is a serious soft tissue infection typically arising from dental infections. It presents with diffuse facial swelling that needs prompt surgical intervention and antibiotic therapy. Early diagnosis is crucial to prevent severe complications from the infection. In the current research, the inclusion and exclusion criteria for prior antibiotic use were intentionally structured to reflect authentic clinical practice while limiting methodological bias in microbial profiling. Patients receiving previous oral antibiotics were included to mirror real-life scenarios where individuals often self-medicate with over-the-counter agents before pursuing emergency care. However, patients who received recent parenteral (intravenous or intramuscular) antibiotic administration were excluded to safeguard microbial flora consistency and eliminate confounding selection pressures. Parenteral antibiotics achieve significantly higher peak tissue concentrations, causing more considerable disruptions to local and systemic microbiota when compared to oral formulations. In contrast, oral antibiotics display subtler shifts and lower bioavailability. This is attributable to the limited amount of active antibiotic components available for absorption, and this consequently reduces their overall impact^33^. This perspective aligns with prior research, such as that reported in^34^. The study mentioned similarly permitted oral antibiotics and excluded parenteral ones to allow maintenance of the analytical reliability.

As for Egypt, such practices are relevant in real life. It was apparent when a survey conducted in Alexandria found that 22% of residents were using antibiotics for viral illnesses like the common cold. Despite educational campaigns, behavioral change remained limited, and widespread oral antibiotic misuse persisted, subtly altering presenting flora in patients without the dramatic effects of injectable antibiotics^15^.

Sample collection during incision and drainage was carried out in the present work. This approach improved the possibility of directly isolating the causative pathogens from infection sites. Additionally, it allows a direct approach to purulent material for culturing and Gram staining, which yields positive results for (91.18%) of cases. This makes it the most recommended way to diagnose the specific pathogen involved^35^. The samples underwent direct transport at 4 °C to the lab. This transport strategy minimizes microbial degradation, which is crucial for reliable culturing outcomes and preserves microorganisms’ viability. The merits of this protocol were strongly supported by a previous review article^36^.

As a result of the present study, odontogenic facial cellulitis is prevalent among both young and older adults, with a slight male predominance (63.64%) over females (36.36%). This gender distribution aligns with global epidemiology reported in^37^, with a male predominance in odontogenic primary facial space infections (female: male ratio 1:1.87, mean age 33.77 ± 13.45 years). Similarly, Słotwińska-Pawlaczyk et al. (2023) and Ekici (2023) reported a higher prevalence of odontogenic facial cellulitis among males. This predominance has been attributed to behavioral and healthcare-seeking differences, with men generally being less likely to seek timely dental care and preventive treatment than women, thereby increasing the likelihood of disease progression before professional intervention^2,37^. At the time of sample collection, most infections commonly originated from lower posterior teeth (70.45%), especially first molars (59.09%). These findings are consistent with established knowledge regarding the anatomical proximity of these teeth to the submandibular and submental spaces^38^. This anatomical relationship explains the high frequency of cellulitis in submandibular and submental regions in both clinical observations and published studies. Such patterns emphasize the clinical need for prompt attention to infections involving lower molars due to their tendency for deeper space involvement and potentially more severe clinical courses^37,39^. In addition to improving statistical power, the anatomical grouping of infection sites for analysis mirrored the clinical realities of contiguous facial space involvement^32^.

For microbiological identification, the study combined standard biochemical tests for the identification of *Staphylococcus aureus* and *Proteus mirabilis.* The choice for the detection of *Staphylococcus aureus* and *Proteus mirabilis.* in the samples from Egyptian patients has an important clinical and epidemiological significance. *Staphylococcus aureus* is a common and dominant pathogen in odontogenic infections, especially facial cellulitis. It is often linked to antibiotic resistance and treatment challenges. Monitoring its prevalence aids in understanding infection dynamics and targeted therapy^40^. On the other side, although *Proteus mirabilis* is less frequently presented in odontogenic infection cases, it is an emerging pathogen in complicated skin and soft tissue infections, including cellulitis, with unique virulence factors and antibiotic susceptibility that influence treatment patterns^41^. In the work presented, the low detection percentages of *Staphylococcus aureus* (18.18%) and *Proteus mirabilis* (9.09%) can be attributed to the inclusion of patients who were on prior oral antibiotic treatment. Previous antibiotic use likely suppressed bacterial growth and reduced detectable bacterial load^42^.

Additionally, conventional culture methods on multiple selective media were carried out together with Gram staining. They are considered as gold standard approaches to secure broad bacterial detection. This study disclosed that most of the cellulitis cases presented were due to single bacterial isolates (68.18%), commonly Gram-positive cocci (73.5%), with a smaller subset showing mixed/ bacillary morphologies.

A comprehensive microbiological approach was adopted in the present study, combining conventional culture on selective and differential media with Gram staining to characterize bacterial isolates. This phenotypic workflow was complemented by 16 S rRNA sequencing of multidrug-resistant isolates, enabling more accurate taxonomic identification of clinically relevant pathogens. Although anaerobic culture techniques were not included in the present study, the applied methodology enabled the identification of a broad range of cultivable aerobic and facultative bacterial species associated with odontogenic facial cellulitis. Consequently, obligate anaerobic organisms may not have been fully represented, and the findings should be interpreted within the scope of the microbiological methods employed^43^. Bertossi et al. (2017) showed close alignment with the results of the present work. 68.18% of the cellulitis cases were due to single microbial infection, with Gram-positive cocci predominating (73.5%). This prevalence underpins the importance of streptococcal species and related Gram-positive organisms in the pathogenesis of odontogenic facial cellulitis. As for the remaining isolates, they showed mixed bacterial morphologies of Gram-negative bacilli and polymicrobial flora. This reflects the typical complex bacterial ecology of oral infections^44^. Such findings confirm previous microbiological investigations that, despite the high frequency of polymicrobial infections, a single predominant pathogen often dominates the clinical scenario of many cellulitis cases. The predominance of Gram-positive cocci supports the current understanding that species like Streptococcus pyogenes and Staphylococcus aureus are major causative organisms. Meanwhile, the presence of mixed flora justifies considering anaerobic and facultative anaerobic bacteria in both diagnostic and therapeutic decision-making. These findings confirm the appropriateness of combined culture and staining techniques to elucidate the microbial profile in odontogenic infections, which substantiates targeted antimicrobial therapy^45^.

In the present research, antimicrobial susceptibility testing was performed via agar well diffusion following CLSI guidelines to ensure verification of results against a well-established clinical standard^24^. Data analysis techniques employed in the study aimed to offer a cautious and clinically relevant interpretation of the burden of antimicrobial resistance. Moreover, these techniques agreed with clinical reasoning, such as age stratification and the conservative grouping of intermediate susceptibility with resistance^29^.

Of particular concern in the antibiotic sensitivity results was the resistance to metronidazole (Flagyl) among all isolates (100%). It is an emerging trend that is increasingly reported worldwide. Metronidazole’s pivotal role as a frontline agent for dental infections and systemic oral diseases is now compromised by overuse and the spread of resistance genes within oral bacterial communities^46^. Similar results were obtained by a study carried out by Sebastian et al., in 2019. The authors reported high resistance rates to metronidazole in aerobic (95%) and anaerobic (86%) bacteria from odontogenic infections^42^. Notably, prior antibiotic use was significantly associated with increased resistance to amoxicillin (26.47%) and clindamycin (41.18%), both of which are commonly used in the management of odontogenic infections. Although resistance to novobiocin was observed among patients with previous antibiotic administration in the present study (65.38%), novobiocin is currently used primarily as a laboratory diagnostic agent rather than a therapeutic antimicrobial^47^. These susceptibility findings have limited clinical relevance and should be interpreted with caution. These findings were emphasized in contemporary past reviews stressing that repeated or inappropriate antibiotic administration exerts selective pressure favoring survival and proliferation of resistant strains and eliminating susceptible ones^48,49^. Furthermore, a significant association between demographic data and antibiotic resistance was identified in the study. For example, the age range dramatically influenced the observed resistance patterns, especially with Amoxicillin and Dalacin. Sensitivity to amoxicillin was higher, especially among the younger age group (19–30 yrs) (89.47%), rather than in (31–60 yrs) (53.33%). The same goes for Dalacin sensitivity. It was greater in the age group (19–30 yrs) (73.68%) than (31–60 yrs) (40%). These age-related trends in antibiotic resistance suggest that both immune reactional changes and differences in antibiotic exposure over a lifetime contribute to the emergence of these patterns. Higher antibiotic resistance is shown in older individuals. This aligns with increased lifetime of antibiotic usage together with higher infection risks in that age group^50^. Moreover, a marked difference was found in the current study regarding prior antibiotic use, with all female participants (16/16, 100%) reporting previous use compared to 71.43% of males (20/28) (*p* = 0.018). This notable disparity highlights the need for further research to understand its underlying causes.

The study implemented 16 S rRNA gene sequencing for multidrug-resistant isolates from the samples. This procedure permitted precise taxonomic identification and evolutionary analysis, and added a high-resolution dimension to phenotypic methods^25^.This molecular technique was applied in a study in 2012 and identified 25 different species or phylotypes of bacteria from various samples of odontogenic infections^51^. Most isolates in the present study showed high sequence identity (> 97%) with reference strains deposited in GenBank, supporting the accuracy of the microbiological identification.

Interestingly, two isolates (isolates 3 and 10) demonstrated less than 90% 16 S rRNA sequence identity with currently available reference sequences and therefore could not be confidently assigned to a recognized bacterial species. According to accepted thresholds for bacterial identification, sequence identities below this level indicate that reliable taxonomic assignment cannot be achieved using partial 16 S rRNA gene analysis alone and may reflect novel taxa, highly divergent lineages, or limitations in currently available sequence databases^52^. Since additional phenotypic characterization, phylogenetic reconstruction, multilocus sequence analysis, or whole-genome sequencing were beyond the scope of the present investigation, no attempt was made to assign these isolates to a putative genus or novel species.

Nevertheless, these unidentified isolates highlight the possibility that the microbial diversity associated with odontogenic facial cellulitis remains incompletely characterized. This observation is consistent with accumulating evidence that the oral microbiome contains numerous poorly characterized or uncultivated microorganisms, many of which remain underrepresented in publicly available databases^53^. Future investigations integrating comprehensive phenotypic characterization with whole-genome sequencing and advanced taxonomic analyses are warranted to determine the phylogenetic position, pathogenic potential, and clinical significance of these unidentified isolates.

The congruence of phylogenetic tree topography from independently robust alignment methods (ClustalW and MUSCLE) strengthened the confidence in the genetic identification and confirmed the close genetic clustering of our isolates with their reference strains **(e.g.**, *Bacillus*, *Enterococcus***).** These dual alignment strategies and model selection based on Bayesian Information Criterion increased the phylogenetic inferences’ robustness, reflecting best practices in microbial genomics^54^. In addition, literature also emphasizes the polymicrobial and the frequent anaerobic nature of odontogenic facial cellulitis, with anaerobes such as *Fusobacterium* and *Prevotella* often reported. While *Staphylococcus*, *Enterococcus*, and *Bacillus* species were the predominant isolates in this study, Similar species distributions were documented in literature, with *Staphylococcus* aureus and various *Streptococci* as frequent culprits^55,56^.

Although *Bacillus* species are generally regarded as ubiquitous environmental microorganisms and potential laboratory contaminants, they have occasionally been reported as opportunistic pathogens in soft tissue wounds and oral infectious lesions, including infected root canals and periodontal lesions^57,58^. This suggests that they may occasionally act as opportunistic pathogens in the oral cavity. In the present study, all clinical specimens were collected and processed under strict aseptic conditions thereby minimizing the likelihood of environmental contamination. Nevertheless, despite these precautions, environmental contamination cannot be completely excluded, particularly in the absence of dedicated contamination controls. Therefore, the predominance of *Bacillus* species observed in the present study should be interpreted with caution and warrant confirmation in future investigations employing larger cohorts, comprehensive anaerobic culture techniques, and rigorous contamination-control measures.

This cross-sectional study design constrains the ability to draw causal conclusions about demographic, resistance relationships and treatment. Nevertheless, the methodological framework combining clinical sampling, conventional and molecular microbiology, and stringent data analysis presents a comprehensive approach to studying odontogenic cellulitis microbiology and antimicrobial resistance. Although the research is limited by sample size and single-center scope, the methods employed allow reliable and clinically relevant observations that can inform effective management strategies.

From a practical standpoint, these findings underscore the necessity of basing empirical therapy for odontogenic cellulitis on updated local resistance profiles. This should be additionally supported by culture and sensitivity results whenever possible. Amoxicillin remains among one of the most useful broad-spectrum antibiotics, yet the rising resistance rates highlight the importance of combination therapy or alternative agents whenever indicated by clinical response or laboratory evidence. Early intervention as surgical drainage remains the mainstay of therapy, with antibiotics serving as a pivotal adjunctive treatment. Moreover, the identification of possibly novel bacterial species within odontogenic infections suggests a need for further microbiome research and ongoing molecular surveillance.

### Study limitations

Although this study provides novel insights into the bacterial profile and antimicrobial susceptibility of odontogenic facial cellulitis in an Egyptian cohort, several limitations should be considered when interpreting the findings. First, its single-center, cross-sectional design, relatively small sample size, and convenience sampling may limit the generalizability of the findings. Second, because anaerobic culture was not performed, the microbiological findings primarily reflect cultivable aerobic and facultative organisms, while obligate anaerobic pathogens may have been underrepresented, therefore, the microbiological findings should be interpreted within the scope of the methods employed. Third, although sterile swabs were used and strict aseptic procedures were followed throughout specimen collection and laboratory processing, the use of swab specimens rather than needle aspiration may have increased the possibility of surface contamination. Therefore, environmental organisms such as *Bacillus* spp. should be interpreted with caution. The antimicrobial susceptibility panel reflected the protocol used during the study period and did not include several antibiotics currently recommended for susceptibility testing of odontogenic infections. In addition, it was not complemented by molecular detection of antimicrobial resistance genes, precluding characterization of the underlying resistance mechanisms. Finally, microbiological analysis was performed at a single pre-treatment time point, preventing assessment of temporal changes in bacterial composition or antimicrobial susceptibility. Future multicenter studies incorporating aspiration sampling, comprehensive aerobic and anaerobic microbiological analyses, expanded antimicrobial resistance characterization, and longitudinal follow-up are warranted to further clarify the microbiology of odontogenic facial cellulitis.

## Conclusion

This cross-sectional study characterizes the aerobic bacterial profile and antimicrobial resistance patterns of odontogenic facial cellulitis in a clinically recruited Egyptian patient cohort, combining conventional microbiology with 16 S rRNA-based molecular identification. Universal metronidazole resistance, age-associated differences in amoxicillin and clindamycin susceptibility, and the identification of two potentially novel bacterial species highlight the complexity of odontogenic infections in the Egyptian context. These findings underscore the urgent need for local antibiotic stewardship policies, regulation of over-the-counter antibiotic dispensing, and ongoing genomic surveillance to optimize empirical therapy and improve clinical outcomes.

## Supplementary Information

Below is the link to the electronic supplementary material.

Supplementary Material 1

## Data Availability

All data generated or analyzed during this study are included in this article.The datasets generated and/or analyzed during the current study are available in the NCBI GenBank repository under the accession numbers; [OR365284](https:/www.ncbi.nlm.nih.gov/nuccore/OR365284) , [OR365495](https:/www.ncbi.nlm.nih.gov/nuccore/OR365495) , [OR365496](https:/www.ncbi.nlm.nih.gov/nuccore/OR365496) , [OR365499](https:/www.ncbi.nlm.nih.gov/nuccore/OR365499) , [OR365504](https:/www.ncbi.nlm.nih.gov/nuccore/OR365504) , [OR365510](https:/www.ncbi.nlm.nih.gov/nuccore/OR365510) , [OR365511](https:/www.ncbi.nlm.nih.gov/nuccore/OR365511) , [OR365514](https:/www.ncbi.nlm.nih.gov/nuccore/OR365514) , [OR365534](https:/www.ncbi.nlm.nih.gov/nuccore/OR365534) , [OR365662](https:/www.ncbi.nlm.nih.gov/nuccore/OR365662) , [OR365663](https:/www.ncbi.nlm.nih.gov/nuccore/OR365663) , [OR365665](https:/www.ncbi.nlm.nih.gov/nuccore/OR365665) .
