# Supplementary material for: Pathogenic bacterial profile and antimicrobial susceptibility patterns in odontogenic facial cellulitis among Egyptian patients: A cross-sectional study
Source: Sci Rep. 2026 Aug 6;16:24321. doi: 10.1038/s41598-026-64016-7 (PMC13448242; doi:10.1038/s41598-026-64016-7)
Supplement: Supplementary file 1 [file 41598_2026_64016_MOESM1_ESM.docx]

**Figure (1. a):** Phylogenetic tree Isolate_1_clustalw (*Bacillus paramycoides* strain MCCC1A04098, 98.88 % identity).

**Figure (1. b):** Phylogenetic tree Isolate_1_muscle (*Bacillus paramycoides* strain MCCC1A04098, 98.88% identity).

**Figure (2. a):** Phylogenetic tree Isolate_2_clustalw (*Bacillus cereus* strain JCM 2152, 99.35% identity).

**Figure (2. b) :** Phylogenetic tree Isolate_2_muscle (*Bacillus cereus strain* JCM 2152, 99.35% identity).

**Figure (3. a):** Phylogenetic tree Isolate_4_clustalw (*Bacillus fungorum 17-SMS-01,* 99.63% identity)

**Figure (3. b):** Phylogenetic tree Isolate_4_muscle (*Bacillus fungorum* 17-SMS-01, 99.63% identity).

**Figure (4. a) :** Phylogenetic tree Isolate_5_clustalw (*Staphylococcus aureus* strain NBRC 100910, 99.25% identity)

**Figure (4. b):** Phylogenetic tree Isolate_5_muscle (*Staphylococcus aureus* strain NBRC 100910, 99.25 % identity).

**Figure (5. a**): Phylogenetic tree Isolate_6_clustalw (*Staphylococcus schweitzeri* strain DSM 28300, 99.40% identity.)

**Figure (5. b):** Phylogenetic tree Isolate_6_muscle (*Staphylococcus schweitzeri* strain DSM 28300, 99.40% identity).

**Figure (6. a):** Phylogenetic tree Isolate_7_clustalw (*Staphylococcus simiae* CCM 7213 = CCUG 51256, 97.20% identity).

**Figure (6. b):** Phylogenetic tree Isolate_7_muscle (*Staphylococcus simiae* CCM 7213 = CCUG 51256, 97.20% identity)

**Figure (7. a):** Phylogenetic tree Isolate_8_clustalw (*Klebsiella pneumoniae* subsp. *ozaenae* strain ATCC 11296, 98.04% identity).

**Figure (7. b):** Phylogenetic tree Isolate_8_muscle (*Klebsiella* *pneumoniae* subsp. *ozaenae* strain ATCC 11296 98.04% identity).

**Figure (8. a):** Phylogenetic tree Isolate_9_clustalw (*Enterococcus faecium* strain NBRC 100485 98.63% identity).

**Figure (8. b) :** Phylogenetic tree Isolate_9_muscle ( *Enterococcus faecium* strain NBRC 100485 98.63% identity).

**Figure (9. a):** Phylogenetic tree Isolate_11_clustalw (*Bacillus licheniformis* DSM 13, 98.44% identity).

**Figure (9. b):** Phylogenetic tree Isolate_11_muscle (*Bacillus licheniformis* DSM 13, 98.44% identity).

**Figure (10. a):** Phylogenetic tree Isolate_12_clustalw (*Enterococcus faecium* LMG 11423 98.09% identity).

**Figure (10. b):** Phylogenetic tree Isolate_12_muscle (*Enterococcus faecium* LMG 11423 98.09% identity).

**Figure (11. a):** Phylogenetic tree Isolate_13_clustalw (*Bacillus Sanguinis* BML-BC004, 98.93% identity).

**Figure (11. b) :** Phylogenetic tree Isolate_13_muscle (*Bacillus sanguinis* BML-BC004, 98.93% identity).

**Figure (12. a) :** Phylogenetic tree Isolate_14_clustalw (*Enterococcus asini*, NBRC 100681, 98.99% identity).

**Figure (12. b) :** Phylogenetic tree Isolate_14_muscle (*Enterococcus asini*, NBRC 100681 98.99% identity)
